# Quantifying right ventricular motion and strain using 3D cine DENSE MRI

**DOI:** 10.1186/1532-429X-13-S1-M3

**Published:** 2011-02-02

**Authors:** Daniel A Auger, Xiaodong Zhong, Ernesta M Meintjes, Frederick H Epstein, Bruce S Spottiswoode

**Affiliations:** 1 University of Cape Town, Cape Town, South Africa; 2 MR R&D Collaborations, Siemens Medical Solutions, Atlanta, GA, USA; 3 University of Virginia, Charlottesville, VA, USA

## Objective

The purpose of this study is to quantify right ventricular (RV) motion and surface strain in normal volunteers using 3D cine DENSE MRI.

## Background

The RV is difficult to image because of its thin wall, asymmetric geometry and complex motion. DENSE is a quantitative MRI technique for measuring myocardial displacement and strain at high spatial and temporal resolutions [1,2]. DENSE encodes tissue displacement directly into the image phase, allowing for the direct extraction of motion data at a pixel resolution. A free-breathing navigator-gated spiral 3D cine DENSE sequence was recently developed [3], providing an MRI technique which is well suited to quantifying RV mechanics.

## Methods

Whole heart 3D cine DENSE data were acquired from two normal volunteers, after informed consent was obtained and in accordance with protocols approved by the University of Virginia institutional review board. The endocardial and epicardial contours were manually delineated to identify the myocardium from surrounding anatomical structures. A 3D spatiotemporal phase unwrapping algorithm was used to remove phase aliasing [4], and 3D Lagrangian displacement fields were derived for all cardiac phases. Midline contours were calculated from the epicardial and endocardial contours, and tissue tracking seed points were defined at pixel spaced intervals. A 3D tracking algorithm was implemented as a direct extension of the 2D tracking algorithm presented in [4], producing midline motion trajectories from which strain was calculated. Tangential 1D strain was calculated in the longitudinal and circumferential cardiac directions. Strain time curves are computed representing the free wall of the RV.

## Results

**Figure 1:**
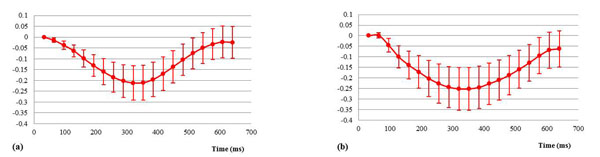
Data shown as mean ± one standard deviation. (a) Circumferential and (b) Longitudinal strain

Figure 1 illustrates the RV free wall mean tangential 1D strain time curves for approximately 3/4 of the cardiac cycle over the apical-mid section of the heart for one volunteer. Results show measurements ranging between -0.1 and -0.25, and further illustrate a greater displacement in the longitudinal direction. Results compare favorably with studies using myocardial tagging and DENSE [5,6].

## Conclusion

This work presents 3D motion tracking and strain quantification of the RV at a previously unattainable spatial resolution.
